# Exosomal miRNAs as Biomarkers of Ischemic Stroke

**DOI:** 10.3390/brainsci13121647

**Published:** 2023-11-27

**Authors:** Anna Maria Ciaccio, Antonino Tuttolomondo

**Affiliations:** Internal Medicine and Stroke Care Ward, Regional Reference Center for Diagnosis and Treatment of Anderson-Fabry Disease, Department of Health Promotion, Maternal and Child Health, Internal Medicine, and Specialty Excellence “G. D’Alessandro” (PROMISE), University of Palermo, 90127 Palermo, Italy; annamaria.ciaccio@unipa.it

**Keywords:** exosome, stroke, miRNA

## Abstract

Exosomes are small lipid bilayer membrane particles released from all living cells into the extracellular environment. They carry several molecules and have a critical role in cell–cell communication under physiological and pathological conditions. In recent decades, exosomes, and especially their cargo, have emerged as a promising tool for several clinical conditions. However, the literature has become increasingly unambiguous in defining the role of exosomes in chronic cerebrovascular diseases. Because they can pass through the blood–brain barrier, they have great potential to reflect intracerebral changes. They can, thus, provide valuable insight into the mechanisms of central nervous system diseases. The purpose of this review is to describe the literature on the role of exosomal miRNA, which represents the most widely investigated exosomal biomarker, in strokes. First, we provide an overview of exosomes, from biology to isolation and characterization. Then, we describe the relationship between exosomes and stroke pathogenesis. Finally, we summarize the human studies evaluating exosomal miRNA biomarkers of stroke. Although the collective literature supports the potential use of exosomal miRNA as biomarkers of ischemic stroke, there are still several limitations hampering their introduction into clinical practice.

## 1. Introduction

Stroke is a major cause of death and disability worldwide, with an increasing incidence in developing countries, according to a report published by the World Health Organization in 2020 [1]. It is classified into two main categories: ischemic and hemorrhagic. Ischemic stroke due to cerebral vascular occlusion is the most common form, while hemorrhagic stroke due to cerebral bleeding accounts for about 12% of cases [2].

Early recognition of stroke is fundamental to prompt treatment and improve patient outcomes. However, early diagnosis is challenging because signs and symptoms are not specific and can overlap with other neurological diseases. Indeed, several neurological and non-neurological conditions can mimic stroke, such as cerebral tumors, traumatic injuries, infections, and epilepsy [3]. To date, stroke diagnosis is mainly based on clinical evaluation and radiological investigations [4]. Brain imaging has a critical role in distinguishing ischemic and hemorrhagic strokes and guiding clinicians to the appropriate treatment [5]. Nevertheless, neuroimaging has some limitations, including costs, contraindications, the need for an experienced radiologist to interpret results, and restricted availability. Thus, there is intensive scientific research to find reliable tools to support clinicians in appropriately managing stroke patients.

Unlike most high-incidence diseases, such as Diabetes and Acute Myocardial Infarction, no specific and sensitive stroke biomarker is currently available in clinical practice. In this field, biomarker identification is hampered by the heterogeneity of the disease, the complexity of the stroke pathophysiology, and the impact of the blood–brain barrier (BBB) on the biomarker’s diffusion in the circulation [6]. The ideal biological fluid for detecting stroke biomarkers would be cerebrospinal fluid (CSF). However, it is unsuitable in emergencies because the CSF collection is invasive and requires specialized personnel. Thus, many efforts are ongoing to detect reliable circulating biomarkers.

**Figure 1:**
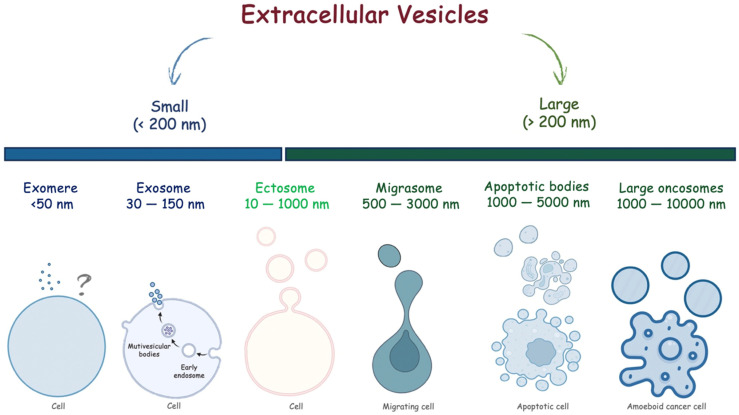
Extracellular vesicles classified according to their sizes.

In the last decade, a role for extracellular vesicles (EVs) has emerged. EVs are small lipid bilayer membrane particles released from all living cells into the extracellular environment [7]. Broadly, EVs can be classified as small, with a diameter less than 200 nm, and large, with a diameter greater than 200 nm. Different subtypes of EVs have been described to date. Specifically, large oncosomes, apoptotic bodies, ectosomes, and migrasomes are large EVs, while exomeres and exosomes are small EVs (Figure 1). Beyond sizes, EVs have different biogenesis [8]. Briefly, large oncosomes result from membrane blebs of amoeboid cancerous cells, apoptotic bodies from membrane blebs of apoptotic cells, migrasomes via migracytosis during cell migration, ectosomes via outward budding of the plasmatic membrane, and exosomes via the endosome–multivesicular body (MVB) pathway followed by fusion to the plasma membrane of cells. Exomere represents the smallest EVs, but the biogenesis mechanisms are not understood yet.

EVs are not simple lipid structures but play an active role in different biological mechanisms, including intracellular homeostasis and intercellular communication. Indeed, they carry various bioactive molecules, such as nucleic acids, lipids, proteins, and metabolites. EVs released from brain cells can cross the BBB and be detected in different biofluids, including blood. Thus, they could provide important information on the status of the cerebral nervous system.

As the knowledge of EVs has grown exponentially since 2000, some researchers have assessed their role as biomarkers of various pathological processes, such as cancer, neurodegenerative diseases, kidney diseases, infections, and cardiovascular diseases, including stroke [9,10,11,12]. Among all EVs, exosomes are the most widely investigated.

The aim of this review is to describe the literature on the role of exosomal miRNA as a biomarker of stroke. First, we provide an overview of the biology of exosomes. Then, we present the relationship between exosomes and the pathogenesis of ischemic stroke. Finally, we summarize the human studies on exosomes as biomarkers of ischemic stroke.

## 2. Exosomes

The discovery of exosomes dates to the 1940s, when Chargaff detected small sedimented “membrane debris” while studying the effect of high-speed centrifugation on the clotting time of human plasma [13]. However, only several decades later, the basis of the current knowledge on sEVs was laid [14,15].

In the 1980s, the era of EV research began. In 1983, Harding et al. and Pan and Jonhstone independently published two scientific articles on reticulocyte maturation, defined as exosomes, the intraluminal vesicles released from cells [16,17]. In the next decade, EVs were thought to be waste disposal mechanisms. The widespread idea was that EVs represented a way to shed obsolete molecules. Only in the early 21st century emerged the hypothesis that EVs could have important physiological functions and be involved in pathological mechanisms, leading to the explosion of scientific interest in EVs [18].

The International Society for Extracellular Vesicles (ISEV) proposed Minimal Information for Studies of Extracellular Vesicles (“MISEV”) guidelines, which were first published in 2014 (MISEV2014) and then updated in 2018 (MISEV2018) [19]. According to ISEV, extracellular vesicle is a generic term referring to “particles naturally released from the cell that is limited by a lipid bilayer and cannot replicate”. Exosomes are a subtype of EVs.

### 2.1. Biogenesis and Function

**Figure 2:**
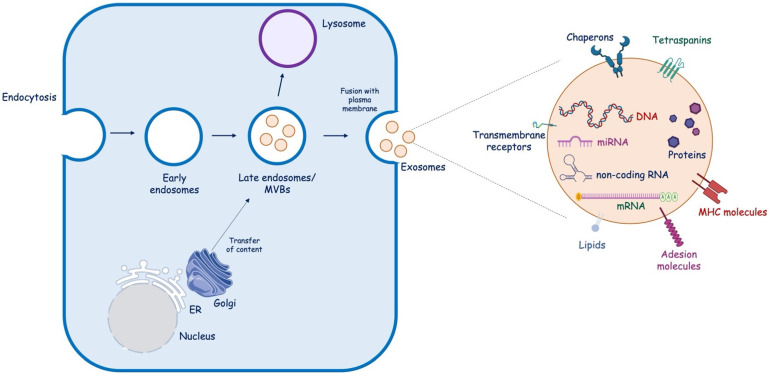
Exosome biogenesis. Endocytosis of the plasma membrane results in the formation of early endosomes, which then mature into late endosomes. Within endosomes, intraluminal vesicles bud off into the lumen, forming MBVs, which can directly fuse with the plasma membrane, releasing exosomes into the extracellular space, or fuse with the lysosome to be degraded. ER, endoplasmic reticulum; MVB, multivesicular bodies; MHC, major histocompatibility complex; miRNA, microRNA; mRNA, messenger.

Exosome biogenesis is complex and involves the endocytic pathway. It occurs in three key steps: (i) invagination of the plasma membrane to generate early endosomes, which then mature into late endosomes; (ii) inward budding of the endosomal membrane to form intraluminal vesicles (ILVs), which then develop into MVBs [20]; and (iii) the fate of MVBs depends on the protein expressed on their surface. Some MVBs act as “delivery trucks”, and their fusion with the plasma membrane leads to the external release of ILVs as exosomes (Figure 2). Alternatively, MVBs could act as “garbage trucks”, and they bind lysosomes for degradation and removal. Exosomes differ in terms of internal components, which could include DNA, non-coding RNAs (nc-RNAs), such as miRNAs (≈22 nucleotides), long ncRNA (>200 nucleotides in length), and circular RNA (a circular molecule that has a covalently closed loop structure, lacking a poly-A tail or 5′→3′ polarity), mRNA, and proteins, and transmembrane molecules, such as chaperons, tetraspanins, lipids, receptors, and major histocompatibility complex class II. The exosomes’ composition denotes their cellular origin and potential biological functions [21].

Exosomes are crucial in cell–cell communication under physiological and pathological conditions. Indeed, they cargo different molecules to target cells. Among these, miRNAs are the most widely investigated due to their pivotal role in regulating gene expression. miRNAs are small, non-coding, single-stranded RNAs with about 19–25 nucleotides [22]. They exert their action by interacting with specific sequences at the 3′ untranslated region (UTR) of the target mRNA, leading to expression repression [23]. miRNAs could also interact with other sequences of the target mRNA, including the 5′ UTR, promoters, and coding sequences. Additionally, under specific conditions, miRNA could promote mRNA transcription [24]. Interestingly, a single miRNA can regulate thousands of target genes [25]. Circulating miRNAs can be detected in different body fluids, including blood, breast milk, saliva, and urine, and they can be secreted by several mechanisms, including passive secretion during necrosis or active release by exosomes [26]. It has been shown that exosomes protect miRNAs from circulating RNA-degrading enzymes, making them more stable [27].

The exosomal miRNA profile differs between health and disease, indicating that different mechanisms regulate their expression. Thus, their use as biomarkers in diseases attracted much attention. Noteworthy, they have several advantages because they are highly stable in vivo and in vitro, and they can be easily collected using non-invasive procedures, making their measurement repeatable; thus, this allows the monitoring of diseases, and, compared to other existing biomarkers, their content reflects the status of the parental cells.

The transfer of exosomal cargo, including miRNA, into targeted cells is mediated with different mechanisms, such as phagocytosis, receptor-mediated endocytosis, and micropinocytosis [28]. Their pivotal role in cell communication has gained much attention from the scientific community, especially in cancer research [29,30]. Some authors assessed their role in other clinical conditions, such as Diabetes and autoimmune diseases [31,32]. In recent decades, the role of exosomes in stroke has also been explored. As they can cross the BBB, they have a great potential to mirror the intracerebral alterations, and, thus, they could provide precious information on the pathological mechanisms within the central nervous system (CNS).

### 2.2. Isolation Methods and Characterization

Accurate separation is the first step in the study and clinical application of exosomes. However, the isolation and purification of exosomes are extremely challenging due to their essential heterogeneity, small size, and low density [33].

**Figure 3:**
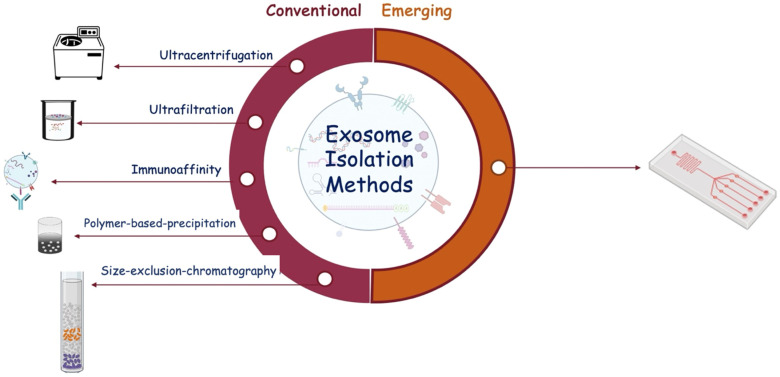
Schematic representation of exosome isolation methods classified as conventional and emerging.

Several methods have been developed to isolate and characterize exosomes from body fluids. We can distinguish between conventional isolation techniques, such as ultracentrifugation, ultrafiltration, immunoaffinity, size exclusion chromatography, and polymer precipitation, and new emerging technologies, such as microfluidic chips (Figure 3).

Ultracentrifugation is the most commonly used method for exosome purification. The principle exploits the differences in density and size between exosomes and other biological material within the sample. It consists of a low-speed centrifugation to remove cells and large vesicles and a high-speed ultracentrifugation to pellet exosomes. Density gradients may remove contaminants. However, ultracentrifugation requires special equipment and specialized professionals; it is time-consuming (>4 h) and operator-dependent; and it has poor repeatability [34,35]. Finally, some of the literature suggests that ultracentrifugation steps can deteriorate exosomes, impairing the downstream analysis of exosome content [36]. Thus, it is not suitable for use in clinical settings.

Ultrafiltration is the easiest method for exosome isolation. It uses a filtering membrane and driving force, such as electric charge, centrifugation, or pressure, to separate exosomes according to molecular size [36]. It does not require special equipment. However, it could be associated with a low recovery rate.

Size exclusion chromatography uses columns containing porous beads, such as Sepharose or Sephadex, to isolate exosomes via gravity or low-speed centrifugation according to molecular size. This method allows for obtaining more purified exosomes, representing an essential issue for exosome analysis [37,38]. Additionally, it is simple and economical for large-scale samples.

Immunoaffinity is based on the antigen-antibody reaction. Specifically, antibodies fixed on a solid surface, such as magnetic beads, recognize exosomal proteins [39]. This technique is characterized by high specificity and isolation purity. Moreover, it isolates specific exosome subclasses by targeting specific exosomal proteins. Nevertheless, it is time-consuming and expensive. Additionally, the elution buffer used to separate antigens, i.e., exosomes, and antibodies may alter the biological function of the collected exosomes.

Polymer-based exosome precipitation uses a polymer, such as protamine and polyethylene, that creates a hydrophobic microenvironment by binding water molecules and, thereby, reducing the solubility of exosomes, allowing them to sediment out of the solution and, consequently, to be collected via low-speed centrifugation [40,41]. This approach is simple, fast, and costless, requiring only a conventional centrifuge. Additionally, it has a high exosome yield, allowing downstream molecular analyses and simultaneous processing of multiple samples. Although it does not require expensive equipment, reagents have high costs. Another disadvantage is the possible protein contamination due to the co-precipitation of soluble non-exosomal proteins [42]. To overcome such issues, some authors proposed a combination with other separative methods, such as size exclusion chromatography [43].

Recently, several manufacturers introduced commercial kits to isolate exosomes based on conventional methods. They are easy to perform, do not require specialized equipment, and allow isolating exosomes from several biological matrices. However, collected exosomes’ purity, quantity, and yield significantly differ among kits [44]. Additionally, they are costly.

Overall, conventional exosome separation methods are widely used but have several technical limitations, including operation complexity, time consumption, large sample volumes, and protein contamination, leading to low isolation efficiency and purity.

In recent decades, new separation technologies have been developed. Among these, microfluidic chips will be expected to be promising tools. Different microfluidics-based methods have been proposed for exosome purification, including physical property-based and immunoaffinity-based microfluidic devices (the review by Chen et al. [44] can be read for more in-depth information on the topic). Microfluidic technologies allow the efficient isolation of exosomes with high throughput capacity and low time consumption. However, some issues, such as the need for advanced equipment and costs, limit their use for large-scale applications [45].

The ideal exosome isolation technique should be easy to perform without complex equipment, rapid, inexpensive, and allow the isolation of exosomes with high throughput, specificity, and low contamination, preserving their integrity and biological activity. To date, no method has satisfied all these requirements. Thus, despite significant advancements in the field of exosome purification, many efforts are mandatory to improve the isolation methods to achieve standardization.

Once isolated, exosomes must be characterized. The characterization is fundamental to making sure that the isolated components are exosomes. The methods for exosome characterization can be divided into two types: (i) methods based on the evaluation of biological characteristics of the exosomes, such as morphology via electron microscopy or size with dynamic light scattering technology and nanoparticle tracking analysis technology; (ii) methods based on the detection of the expression of exosomal proteins using Western blot, ELISA, or flow cytometry. CD9, CD63, and CD81 are tetrasparins, i.e., membrane proteins, most commonly used to characterize exosomes [33].

## 3. Ischemic Stroke

Ischemic stroke is a clinical condition due to a partial or total cerebral artery occlusion disrupting the blood supply to a brain region. The lack of cerebral perfusion leads to the necrosis of the cells in the center of the area supplied by the occluded vessel, resulting in an “*ischemic core*”, and the suffering of the cerebral cells of the adjacent areas, which constitute the “*ischemic penumbra*”. The latter is functionally silent but metabolically active.

Brain tissue is extremely sensitive to ischemia, so even brief ischemic periods in neurons can trigger a complex sequence of events that ultimately may culminate in cellular death. Timely therapeutic intervention prevents the expansion of the damage from the “ischemic core” to the “ischemic penumbra”, thus preserving a greater portion of parenchyma and neuronal elements and reducing residual disability after stroke. Therefore, early recognition of ischemic stroke and immediate intervention are critical to improving patient prognosis and outcome.

### Ischemic Stroke Pathogenesis

The pathophysiological mechanisms underlying cerebral ischemia follow a well-defined temporal sequence, which can be divided into three phases: (1) within a few hours of ischemia, as a result of reduced blood flow and lack of oxygen and nutrients to the brain tissue, energy depletion leads to excitotoxicity and depolarization in the peri-infarct area; (2) over the course of a few days, the pro-inflammatory cytokines from damaged neuronal cells draw macrophages and monocytes into the “ischemic penumbra” and trigger brain inflammation and oxidative stress; and (3) inflammation and reactive oxygen species (ROS) induce necrosis and apoptosis of brain cells through direct damage to the mitochondrion and DNA that lasts for days and weeks. Furthermore, oxidative stress and inflammation induce the progressive destruction of the blood–brain barrier, further aggravating brain damage and the progression of cerebral ischemia [46,47,48,49].

Among all molecular mechanisms underpinning ischemic stroke, neuroinflammation has a critical role [50]. Within a few minutes of the ischemic event, microglia, which represent the component of the innate immune system resident in the central nervous system and constitute approximately 5–20% of all glial cells, undergo activation, leading to their morphological, phenotypic, and functional changes. From a morphological point of view, activated microglial cells have an amoeboid appearance, characterized by some ramifications, like macrophages. Activated microglial cells resemble macrophages in their functions, acquiring the ability to expose antigens, release various pro-inflammatory cytokines and chemokines, and secrete extracellular matrix metalloproteinases contributing to the blood–brain barrier injury. This process could promote the early passage of leukocytes from the systemic circulation to the brain parenchyma, resulting in an increased concentration of pro-inflammatory mediators that worsen the brain injury due to ischemia.

Astrocytes are also a leading actor in ischemic stroke pathogenesis. They are the most abundant glial cells in the CNS. Under physiological conditions, they play critical functions, including regulating plasticity and synaptic transmission, maintaining homeostasis, and controlling cerebral blood flow [51]. After a stroke injury, astrocytes switch from resting to a reactive state [52]. The resulting reactive astrocytes change their gene expression, morphology, proliferation, and function. Noteworthy, the literature suggests that reactive astrocytes have both protective and harmful effects, representing a double-edged sword. Specifically, during ischemic stroke, reactive astrocytes often play different roles according to the type, degree, and location of ischemia and different time points after injury. On the one hand, active astrocytes participate in post-ischemic recovery by alleviating oxidative stress, releasing neurotrophic factors, reducing cerebral edema, protecting neurons, and reducing infarct volume [51]. In contrast, astrocytes contribute to the injury progression by promoting excitotoxicity and an excessive inflammatory response. Although the exact role of astrocytes is still debated, reactive astrocytes play an essential role in ischemic stroke pathogenesis.

## 4. Exosomes in Ischemic Stroke 

During brain injury, exosomes can be released by different cell types within the CNS and could have a prominent role in brain remodeling post-stroke.

Astrocytes represent the most abundant glial cells within the CNS and play a critical role in cerebral homeostasis [53]. Under physiological and pathological conditions, astrocytes release sEVs. Astrocyte-derived sEVs contain different biological molecules, including DNA, miRNA, and proteins, but the composition varies according to the stimuli. Interestingly, under physiological conditions, astrocyte-derived sEVs are enriched with neuroprotective and neurotrophic elements as well as molecules to stimulate neurite outgrowth, synaptic transmission, and neuronal survival. Increasing evidence suggests that astrocytes are activated during cerebral ischemia and could secrete exosomes to protect the CNS. Pei et al. showed that astrocyte-derived exosomes inhibit autophagy and improve neuronal viability in ischemic stroke models [54]. Additionally, astrocyte-derived exosomes contain miRNAs, such as miR-34c and miR-361, that can, respectively, protect neurons and prevent nerve damage after cerebral ischemia [55,56]. Xin et al., in an in vitro model, showed that oxygen–glucose depletion (OGD) in astrocyte-derived exosomes enriched miR-133b-induced neuron outgrowth post-stroke [57]. Pei et al. revealed that miR-190b shuttled via exosomes is involved in preventing OGD-induced autophagy and inhibiting neuronal apoptosis [54]. Beyond miRNA, astrocytes also shuttle prion proteins, which could ameliorate neuronal survival. Finally, astrocyte-derived sEVs can also deliver apolipoprotein D, a classical neuroprotective protein, to neurons, improving neuronal survival [58].

The CNS is regarded as an immune-privileged organ in which adaptive immunity and inflammation are highly controlled to protect neural cells from possible immune response-mediated injury [59]. Microglia are the primary resident innate immune cells of the CNS, and upon activation, they participate in immunoregulation. They are highly dynamic cells that can vary in morphology, from ramified to amoeboid, and phenotype, from pro-inflammatory M1 to anti-inflammatory M2 types [60]. The M1 phenotype secretes cytokines such as interleukin-6 (IL-6) and tumor necrosis factor-α (TNFα), nitric oxide (NO), and reactive oxygen species (ROS). In contrast, the M2 phenotype releases anti-inflammatory biomarkers, such as IL-10, transforming growth factor-β (TGF-β), and IL-4, and exerts neuroprotective effects, including cellular debris removal, angiogenesis promotion, and repair mechanisms. During ischemic stroke, microglia have a “double-edged sword” function, switching between pro- and anti-inflammatory phenotypes [61]. Specifically, it has been shown that the M2 phenotype is prevalent during the early stage of ischemic stroke and then progressively switches to the M1 phenotype. Several mechanisms regulate microglia polarization [62]. The content of sEVs derived from microglia varies based on the M1/M2 phenotypes. Song et al. showed that microglia-derived sEVs intravenously injected into mouse brains immediately after middle cerebral artery occlusion reduced ischemic brain injury and promoted neuronal survival via exosomal miR-124 and its downstream target USP14 [63]. Similarly, Zhang et al. found that miRNA-137 targeting the Notch-1 gene participated in the neuroprotective effect in OGD-treated neurons and transient middle cerebral artery occlusion (TMCAO)-treated mice [64]. On the other hand, some sEV-associated factors could worsen the ischemic injury. Xie et al. showed that exosomal shuttled miR-424-5p induces brain microvascular endothelial cell injury targeting the FGF2-mediated STAT3 signaling pathway [65].

A crosstalk between microglia and neurons has been described both under physiological and pathological conditions, including stroke. Emerging data indicate neurons can release sEV, guiding post-stroke recovery [66]. It has been suggested that neuron-derived sEVs could regulate microglial activation and function, promoting neuronal survival during ischemic stroke. Some authors showed that neuron-derived sEV could induce M2-type microglia polarization [67,68]. Neuron sEV-derived miR-98 emerged as an intercellular signal mediating neurons and microglia communication during brain remodeling after ischemic stroke. Yang et al. revealed that miR-98 regulates microglial phagocytosis by targeting platelet-activating factor receptor (PAFR), which is involved in neuronal pyroptosis during ischemia/reperfusion (I/R) and thus plays neuroprotection in stroke [69,70].

Neurons also communicate with astrocytes through sEV. Oligodendrocytes, neural tube-derived cells producing myelin, a lipid-rich membrane that wraps axons, providing insulation and metabolic support, have been proven to release sEV, which is captured by neurons in in vitro models [71,72]. Fröhlich et al., in an in vitro model of ischemia, showed that oligodendrocyte-derived sEV promoted neuronal survival [73].

Finally, brain microvascular endothelial cells secrete sEVs, which contain multiple factors with a critical role in protecting neurons under hypoxia through several mechanisms, including apoptosis inhibition, angiogenesis, and neurogenesis promotion.

**Figure 4:**
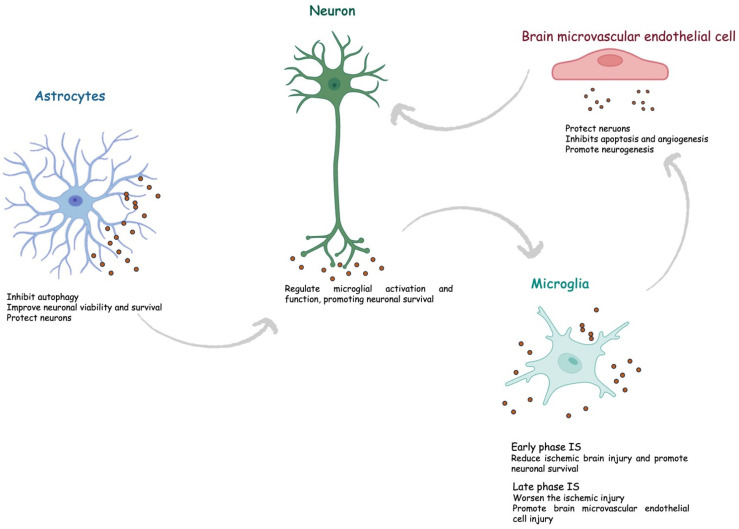
Role of exosomes in ischemic stroke. IS, ischemic stroke.

Overall, cerebral exosomes protect the CNS after cerebral ischemia and contribute to post-ischemic recovery (Figure 4).

## 5. Exosomal miRNAs as Biomarkers of Ischemic Stroke

Biomarkers are defined as a characteristic that is objectively measured and evaluated as an indicator of normal biological processes, pathogenic processes, or pharmacological responses to a therapeutic intervention, according to the U.S. National Institutes of Health working group [69,74]. There is intensive research to find reliable biomarkers to guide clinicians to early and differential diagnosis, prognosis, and monitoring. To date, a plethora of biomarkers of brain injury have been assessed, and the list continues to increase, but, so far, no stroke biomarker is available in clinical practice. The ideal biomarker should have high sensitivity and specificity, be cost-effective, and be easy to measure in emergencies. The biological matrix should be readily accessible and not invasive. The ideal biological fluid for assessing stroke biomarkers should be cerebrospinal fluid (CSF) because it is within the CNS and, thus, reflects the pathological alterations during an acute event. However, it has important limitations, including the invasiveness of the collection procedure and the correlated risks, making it unsuitable in clinical practice.

The detection of circulating stroke biomarkers is hampered by the BBB, a dynamic interface between the peripheral circulation and CNS, limiting the transition of biomarkers from CSF to blood. Exosomes have great potential because they can cross the BBB and, consequently, can be easily measured in blood. Recently, some authors assessed the possible role of exosomes as stroke biomarkers. Patients with ischemic stroke have cargo exosomes different from controls. miRNAs, which represent the most investigated content of exosomes, could provide precious information.

To date, most studies have been performed in animal models and in vitro. Noteworthy, despite stroke being a major risk factor for stroke, most studies have been performed on young animals, limiting the reliability of the findings of these studies [75]. Less evidence comes from human studies. In this section, we summarize the studies exploring the role of exosomal miRNAs as biomarkers of ischemic stroke.

Zhou et al., in an observational study, found higher expression levels of miR-134 in stroke patients than controls [76]. The receiver operating characteristic (ROC) curve analysis showed good performance for diagnosing stroke with an area under the curve (AUC) of 0.834 (95% confidence interval, 0.88–0.97). Additionally, they found a significant correlation between the expression of exosomal miR-134, the National Institutes of Health Stroke Scale (NIHSS) score, and the infarct volume. Also, increased levels of miR-134 in stroke patients were associated with a poor prognosis. Thus, the authors conclude that miR-134 could represent a diagnostic and prognostic biomarker of ischemic stroke.

Similarly, Chen et al., in a retrospective case–control study, showed that increased levels of exosomal miR223 were associated with acute ischemic stroke [77]. The ROC curve analysis showed good diagnostic performance of miR-223 with an AUC of 0.859, a sensitivity of 84.0%, and a specificity of 78.8%. Also, exosomal miR-223 expression is positively correlated with the NIHSS score and poor outcomes in stroke patients. Thus, the authors concluded that exosomal miR-223 could be a reliable biomarker of ischemic stroke.

Jiang et al. performed a bioinformatic analysis using the GEO database to explore the association between exosomal miRNA expression and ischemic stroke [78]. They found that three miRNAs, namely hsa-miR-15b-5p, hsa-miR-184, and hsa-miR-16-5p, were highly expressed in the exosomes of patients with ischemic stroke. Thus, these miRNAs could serve as diagnostic biomarkers.

Ji et al. showed that the levels of exosomal miR-9 and miR-124 were significantly elevated in the serum of patients with ischemic stroke compared to controls and correlated with both NIHSS scores and the infarct volume [79]. They also showed that miR-9 and miR-124 could be sensitive biomarkers for diagnosing ischemic stroke with an AUC of 0.8026 (95% CI: 0.7235–0.8816) and 0.6976, respectively. Similarly, Qi et al. assessed the performance of serum exosomal miR-124-3p as a diagnostic biomarker of ischemic stroke [80]. They showed that the expression of miR-124-3p significantly increased in ischemic stroke and was negatively correlated with pro-inflammatory cytokines, including IL1β, IL6, and TNF-α, and the severity of disease. Serum exosomal miR-124-3p had high sensitivity and accuracy in diagnosing ischemic stroke. The authors also found that the overexpression of miR-124-3p mitigated inflammation in murine LPS-induced BV2 microglial cells via regulating p38 MAPK, Erk1/2, and PI3K/Akt signaling pathways. Overall, these findings support the use of miR-124-3p as a diagnostic and predictive marker for early-stage acute ischemic stroke.

Interestingly, Kalani et al. found that a subset of miRNA could differentiate between ischemic and hemorrhagic strokes [81]. Specifically, they found increased expression of miR-27b-3p and miR-146b-5p in ischemic strokes compared to hemorrhagic strokes.

On the contrary, levels of some exosomal miRNA could be decreased in patients with ischemic stroke. Song et al. found lower levels of miR-152-3p in the serum of stroke patients than controls, and the decrease was significantly related to the severity of endothelial injury [82]. Moreover, ROC curve analysis displayed that the AUC of the exosomal miR-152-3p level was 0.935, suggesting that it could be a reliable stroke biomarker.

Wang et al. revealed the role of serum exosomal miR-328-3p as a predictor of short-term prognosis in patients with stroke, with an odd ratio of 5.276 [83]. Similarly, He et al. demonstrated the prognostic role of exosomal miRNAs in acute ischemic stroke [84]. They measured the plasma levels of exosomal miR-125b-5p, miR-15a-3p, miR-15a-5p, and miR-206 24 h after thrombolysis with or without endovascular treatment in 94 patients. The authors found that miR-125b-5p and miR-206 levels were correlated with NIHSS scores and cerebral infarction volumes. Additionally, miR-125b-5p concentrations were significantly increased in patients with an unfavorable outcome. Thus, miR-125b-5p and miR-206 may be considered prognostic biomarkers of ischemic stroke.

Interestingly, some authors did not simply evaluate the different miRNA expressions between patients and controls but explored the possible differences among various stroke subtypes.

Ischemic stroke spans a temporal continuum from hyperacute (<6 h) to acute (1–3 and 4–7 days), subacute (8–14 days), and recovery (>14 days) phases [85]. Wang et al. explored the role of plasma exosomal microRNA-21-5p and microRNA-30a-5p in the different phases of ischemic stroke [86]. They found that miR-21-5p is higher in patients in the subacute and recovery phases than in controls. miR-30a-5p was increased in the hyperacute phase but decreased in the acute phase compared to controls. In the acute phase, both miRNAs were lower than in the hyperacute phase. The ROC curve analysis showed a good accuracy of miR-30a-5p for detecting the hyperacute phase, with an AUC of 0.826. Also, Li et al. evaluated the role of two plasma exosomal miRNAs according to the different phases of ischemic stroke [87]. The authors found that in the subacute phase, miR-422a and miR-125b-2-3p levels significantly decreased compared to both the controls and the acute phase. In the acute phase, miR-422a levels were increased as compared to controls. ROC analysis revealed good performance for miR-422a and miR-125b-2-3p in the subacute phase, with an AUC of 0.971 and 0.889, respectively, and miR-422a in the acute phase, with an AUC of 0.769. Taken together, these findings pave the way for new avenues for using miRNAs to classify stroke and, consequently, provide helpful information to guide appropriate treatment.

Beyond the timely classification, several classification systems have been proposed and are currently used in clinical practice [36]. Trial of Org 10172 in Acute Stroke Treatment (TOAST) classification categorizes stroke patients, based on the etiology, into five subtypes, namely large artery atherosclerosis (LAA), cardioembolism (CE), small artery occlusion (SAO), stroke of other determined cause (SOC), and stroke of undetermined cause [88]. Among these, LAA is the most common subtype. Niu et al. explored the exosomal miRNA profile in patients with ischemic stroke grouped according to TOAST classification [27]. The authors showed that four miRNAs, miR-369-3p, miR-493-3p, miR-379-5p, and miR1296-5p, could reliably identify LAA. Moreover, the combination of three miRNAs improved the accuracy of the single for diagnosing LAA. Finally, the authors found that miR-493-3p and miR-1296-5p were negatively correlated with the NIHSS score. Interestingly, the authors also compared exosomal miRNAs with their counterparts in plasma. They did not find any correlation between exosomal and plasmatic miRNAs, supporting the protective effect of exosomes on miRNAs. Also, van Kralingen et al. assessed the miRNA profile expression in stroke subtypes according to TOAST classification [89]. First, they found that exosomal miRNA-17-5p, miRNA-20b-5p, miRNA-27b-3p, and miRNA-93-5p were significantly increased in patients with ischemic stroke compared to controls, with SAO patients showing the highest levels. Noteworthy, in this study, controls were not healthy subjects as in most studies, but patients with stroke mimic diseases. This represents a strength of the study, conferring greater clinical relevance to the investigated miRNAs as biomarkers of ischemic stroke.

Otero-Ortega et al. explored the role of exosomal miRNAs in ischemic stroke, classified according to their topography, subcortical and cortical–subcortical involvement [90]. Patients with cortical–subcortical ischemic stroke had decreased levels of miR-15a, miR-424, miR-100, and miR-339 compared with subcortical ischemic stroke, and miR-339, miR-100, miR-199a, miR-369a, miR-424, and miR-15a levels were lower than healthy controls. Thus, this study revealed significant differences in miRNA profiles according to stroke topography.

Table 1 summarizes the characteristics of the main human studies investigating the role of exosomal miRNA as a biomarker of ischemic stroke. As is evident from the literature, there is high heterogeneity among miRNAs investigated in the different studies, making it difficult to identify a unique candidate biomarker to introduce in clinical practice. Only miR124 has been evaluated by different authors achieving exciting findings. Thus, future research should test the potential to translate into clinical practice the use of miR124. 

## 6. Conclusions

Stroke is one of the leading causes of death worldwide, but early diagnosis is still challenging, and no reliable biomarker has been identified to date.

Following stroke, exosomes can be secreted by several cerebral cells, cross the BBB, and be detected in peripheral blood. Since a lipid bilayer structure protects them from circulating ribonucleases, exosomal miRNAs are stable and resistant to degradation. Therefore, they have great potential as noninvasive biomarkers reflecting the pathological alterations within the CNS.

In recent decades, there has been a burgeoning interest in exosomal miRNAs as stroke biomarkers. In this article, we describe the main human studies evaluating the performance of exosomal miRNAs in ischemic stroke.

Significant limitations to introducing exosomal miRNA in real-world clinical practice must be mentioned. First, the methods to isolate exosomes are quite complex, and, to date, there is no standardization, making it difficult to compare and reproduce results, as also assessed by the high heterogeneity among studies [91]. It is critical to ensure standardized and reproducible methods for clinical translation. Additionally, exosomal miRNA evaluation is time-consuming, expensive, and requires specialized personnel and laboratories.

Another gap facing the field of exosomal miRNA biomarker discovery is related to the potential confounding factors affecting miRNA expression. Indeed, there is a considerable body of the literature on how miRNA expression may depend on individual factors, such as sex, age, body mass index, diet, exercise, and comorbidities, which could influence findings [92,93,94,95,96,97,98]. However, most studies did not consider the potential influence of such factors on exosomal miRNA expression.

Finally, most of these studies have largely assessed candidate miRNAs in relatively small study populations. Accordingly, it is mandatory to perform large-scale population studies to confirm the preliminary findings.

Despite the initial strong enthusiasm for the potential clinical application of exosomal miRNAs, several questions must be addressed. Thus, the road seems paved, but many efforts are needed before it becomes viable.

## Figures and Tables

**Table 1:** Studies on exosome as diagnostic biomarker of ischemic stroke.

Authors	Study Population	Time Sample Collection of Stroke Onset	Exosomal miRNA	AUC	Sensitivity	Specificity
Zhou et al. [75]	50 patients and 50 controls	Within 24 h	miR-134	0.834 (0.88–0.97)	75.3%	72.8%
Chen et al. [76]	50 patients and 33 controls	Within 72 h	miRNA-223	0.859	84%	78.8%
Ji et al. [78]	65 patients and 66 controls	NA	miR-9 and miR-124	miR-9: 0.8026 (0.7235–0.8816) miR-124: 0.6976 (0.6506–0.7895)	NA	NA
Kalani et al. [80]	21 patients with ischemic stroke and 36 patients with hemorrhagic stroke	Within 24 h	miR-27b-3p and miR-146b-5p	NA	NA	NA
Qi et al. [79]	10 patients and 10 controls	At 2 h, 4 h, and 6 h	miR-124-3p	At 2 h: 0.81 At 4 h: 0.90 At 6 h: 0.94	NA	NA
Song et al. [81]	93 patients and 70 controls	NA	miR-152-3p	0.935 (0.826–0.998)	92.54%	94.19%

AUC, area under the curve; NA, information not available.

## Data Availability

Not applicable.

## References

[1] https://www.emro.who.int/health-topics/stroke-cerebrovascular-accident/index.html.

[2] Unnithan A.K.A., Das J.M., Mehta P. (2023). Hemorrhagic Stroke.

[3] Makris K., Haliassos A., Chondrogianni M., Tsivgoulis G. (2018). Blood biomarkers in ischemic stroke: Potential role and challenges in clinical practice and research. Crit. Rev. Clin. Lab. Sci..

[4] Mead G.E., Sposato L.A., Sampaio Silva G., Yperzeele L., Wu S., Kutlubaev M., Cheyne J., Wahab K., Urrutia V.C., Sharma V.K. (2023). A systematic review and synthesis of global stroke guidelines on behalf of the World Stroke Organization. Int. J. Stroke.

[5] Dagonnier M., Donnan G.A., Davis S.M., Dewey H.M., Howells D.W. (2021). Acute Stroke Biomarkers: Are We There Yet?. Front. Neurol..

[6] Reymond S., Vujić T., Sanchez J.C. (2022). Neurovascular Unit-Derived Extracellular Vesicles: From Their Physiopathological Roles to Their Clinical Applications in Acute Brain Injuries. Biomedicines.

[7] Yokoi A., Ochiya T. (2021). Exosomes and extracellular vesicles: Rethinking the essential values in cancer biology. Semin. Cancer Biol..

[8] Théry C., Witwer K.W., Aikawa E., Alcaraz M.J., Anderson J.D., Andriantsitohaina R., Antoniou A., Arab T., Archer F., Atkin-Smith G.K. (2018). Minimal information for studies of extracellular vesicles 2018 (MISEV2018): A position statement of the International Society for Extracellular Vesicles and update of the MISEV2014 guidelines. J. Extracell. Vesicles.

[9] Lim W.Q., Michelle Luk K.H., Lee K.Y., Nurul N., Loh S.J., Yeow Z.X., Wong Q.X., Daniel Looi Q.H., Chong P.P., How C.W. (2023). Small Extracellular Vesicles’ miRNAs: Biomarkers and Therapeutics for Neurodegenerative Diseases. Pharmaceutics.

[10] Delrue C., De Bruyne S., Speeckaert R., Speeckaert M.M. (2023). Urinary Extracellular Vesicles in Chronic Kidney Disease: From Bench to Bedside?. Diagnostics.

[11] Wang H., Ye X., Spanos M., Wang H., Yang Z., Li G., Xiao J., Zhou L. (2023). Exosomal Non-Coding RNA Mediates Macrophage Polarization: Roles in Cardiovascular Diseases. Biology.

[12] Ghosh S., Dhar R., Gurudas Shivji G., Dey D., Devi A., Jha S.K., Adhikari M.D., Gorai S. (2023). Clinical Impact of Exosomes in Colorectal Cancer Metastasis. ACS Appl. Bio Mater..

[13] Chargaff E. (1945). Cell structure and the problem of blood coagulation. J. Biol. Chem..

[14] Wolf P. (1967). The nature and significance of platelet products in human plasma. Br. J. Haematol..

[15] Nunez E.A., Wallis J., Gershon M.D. (1974). Secretory processes in follicular cells of the bat thyroid. 3. The occurrence of extracellular vesicles and colloid droplets during arousal from hibernation. Am. J. Anat..

[16] Harding C., Heuser J., Stahl P. (1983). Receptor-mediated endocytosis of transferrin and recycling of the transferrin receptor in rat reticulocytes. J. Cell Biol..

[17] Pan B.T., Johnstone R.M. (1983). Fate of the transferrin receptor during maturation of sheep reticulocytes in vitro: Selective externalization of the receptor. Cell.

[18] Couch Y., Buzàs E.I., Di Vizio D., Gho Y.S., Harrison P., Hill A.F., Lötvall J., Raposo G., Stahl P.D., Théry C. (2021). A brief history of nearly EV-erything—The rise and rise of extracellular vesicles. J. Extracell. Vesicles.

[19] Lötvall J., Hill A.F., Hochberg F., Buzás E.I., Di Vizio D., Gardiner C., Gho Y.S., Kurochkin I.V., Mathivanan S., Quesenberry P. (2014). Minimal experimental requirements for definition of extracellular vesicles and their functions: A position statement from the International Society for Extracellular Vesicles. J. Extracell. Vesicles.

[20] Huotari J., Helenius A. (2011). Endosome maturation. EMBO J..

[21] Gao Y., Qin Y., Wan C., Sun Y., Meng J., Huang J., Hu Y., Jin H., Yang K. (2021). Small Extracellular Vesicles: A Novel Avenue for Cancer Management. Front. Oncol..

[22] Lee R.C., Feinbaum R.L., Ambros V. (1993). The *C. elegans* heterochronic gene lin-4 encodes small RNAs with antisense complementarity to lin-14. Cell.

[23] Ha M., Kim V.N. (2014). Regulation of microRNA biogenesis. Nat. Rev. Mol. Cell Biol..

[24] Dharap A., Pokrzywa C., Murali S., Pandi G., Vemuganti R. (2013). MicroRNA miR324-3p induces promoter-mediated expression of RelA gene. PLoS ONE.

[25] O’Brien J., Hayder H., Zayedò Y., Peng C. (2018). Overview of MicroRNA Biogenesis, Mechanisms of Actions, and Circulation. Front. Endocrinol..

[26] Nik Mohamed Kamal N.N.S.B., Shahidan W.N.S. (2020). Non-Exosomal and Exosomal Circulatory MicroRNAs: Which Are More Valid as Biomarkers?. Front. Pharmacol..

[27] Niu M., Li H., Li X., Yan X., Ma A., Pan X., Zhu X. (2021). Circulating Exosomal miRNAs as Novel Biomarkers Perform Superior Diagnostic Efficiency Compared with Plasma miRNAs for Large-Artery Atherosclerosis Stroke. Front. Pharmacol..

[28] Kalluri R., LeBleu V.S. (2020). The biology, function, and biomedical applications of exosomes. Science.

[29] Słomka A., Kornek M., Cho W.C. (2022). Small Extracellular Vesicles and Their Involvement in Cancer Resistance: An Up-to-Date Review. Cells.

[30] Feng L., Guo L., Tanaka Y., Su L. (2022). Tumor-Derived Small Extracellular Vesicles Involved in Breast Cancer Progression and Drug Resistance. Int. J. Mol. Sci..

[31] Nezhad Nezhad M.T., Rajabi M., Nekooeizadeh P., Sanjari S., Pourvirdi B., Heidari M.M., Veradi Esfahani P., Abdoli A., Bagheri S., Tobeiha M. (2022). Systemic lupus erythematosus: From non-coding RNAs to exosomal non-coding RNAs. Pathol. Res. Pr..

[32] Sufianov A., Kostin A., Begliarzade S., Kudriashov V., Ilyasova T., Liang Y., Mukhamedzyanov A., Beylerli O. (2023). Exosomal noncoding RNAs as a novel target for diabetes mellitus and its complications. Noncoding RNA Res..

[33] Lin S., Yu Z., Chen D., Wang Z., Miao J., Li Q. (2020). Progress in Microfluidics-Based Exosome Separation and Detection Technologies for Diagnostic Applications. Small.

[34] Zhang P., Zhou X., He M., Shang Y., Tetlow A.L., Godwin A.K., Zeng Y. (2019). Ultrasensitive detection of circulating exosomes with a 3D-nanopatterned microfluidic chip. Nat. Biomed. Eng..

[35] Tauro B.J., Greening D.W., Mathias R.A., Ji H., Mathivanan S., Scott A.M., Simpson R.J. (2012). Comparison of ultracentrifugation, density gradient separation, and immunoaffinity capture methods for isolating human colon cancer cell line LIM1863-derived exosomes. Methods.

[36] Chen P.H., Gao S., Wang Y.J., Xu A.D., Li Y.S., Wang D. (2012). Classifying Ischemic Stroke, from TOAST to CISS. CNS Neurosci. Ther..

[37] Guan S., Yu H., Yan G., Gao M., Sun W., Zhang X. (2020). Characterization of Urinary Exosomes Purified with Size Exclusion Chromatography and Ultracentrifugation. J. Proteom. Res..

[38] Oeyen E., Van Mol K., Baggerman G., Willems H., Boonen K., Rolfo C., Pauwels P., Jacobs A., Schildermans K., Cho W.C. (2018). Ultrafiltration and size exclusion chromatography combined with asymmetrical-flow field-flow fractionation for the isolation and characterisation of extracellular vesicles from urine. J. Extracell. Vesicles.

[39] Ruivo C.F., Adem B., Silva M., Melo S.A. (2017). The Biology of Cancer Exosomes: Insights and New Perspectives. Cancer Res..

[40] Niu Z., Pang R.T.K., Liu W., Li Q., Cheng R., Yeung W.S.B. (2017). Polymer-based precipitation preserves biological activities of extracellular vesicles from an endometrial cell line. PLoS ONE.

[41] Moon S., Shin D.W., Kim S., Lee Y.S., Mankhong S., Yang S.W., Lee P.H., Park D.H., Kwak H.B., Lee J.S. (2019). Enrichment of Exosome-Like Extracellular Vesicles from Plasma Suitable for Clinical Vesicular miRNA Biomarker Research. J. Clin. Med..

[42] Witwer K.W., Buzás E.I., Bemis L.T., Bora A., Lässer C., Lötvall J., Nolte-’t Hoen E.N., Piper M.G., Sivaraman S., Skog J. (2013). Standardization of sample collection, isolation and analysis methods in extracellular vesicle research. J. Extracell. Vesicles.

[43] Szatanek R., Baran J., Siedlar M., Baj-Krzyworzeka M. (2015). Isolation of extracellular vesicles: Determining the correct approach (Review). Int. J. Mol. Med..

[44] Chen S., Sun F., Qian H., Xu W., Jiang J. (2022). Preconditioning and Engineering Strategies for Improving the Efficacy of Mesenchymal Stem Cell-Derived Exosomes in Cell-Free Therapy. Stem Cells Int..

[45] Liu W.Z., Ma Z.J., Kang X.W. (2022). Current status and outlook of advances in exosome isolation. Anal. Bioanal. Chem..

[46] Kuriakose D., Xiao Z. (2020). Pathophysiology and Treatment of Stroke: Present Status and Future Perspectives. Int. J. Mol. Sci..

[47] Tuttolomondo A. (2023). Immunoinflammatory Background of Neuronal Damage in Stroke. Int. J. Mol. Sci..

[48] Pacinella G., Ciaccio A.M., Tuttolomondo A. (2022). Endothelial Dysfunction and Chronic Inflammation: The Cornerstones of Vascular Alterations in Age-Related Diseases. Int. J. Mol. Sci..

[49] Puleo M.G., Miceli S., Di Chiara T., Pizzo G.M., Della Corte V., Simonetta I., Pinto A., Tuttolomondo A. (2022). Molecular Mechanisms of Inflammasome in Ischemic Stroke Pathogenesis. Pharmaceuticals.

[50] Khoshnam S.E., Winlow W., Farzaneh M., Farbood Y., Moghaddam H.F. (2017). Pathogenic mechanisms following ischemic stroke. Neurol. Sci..

[51] Shen X.Y., Gao Z.K., Han Y., Yuan M., Guo Y.S., Bi X. (2021). Activation and Role of Astrocytes in Ischemic Stroke. Front. Cell Neurosci..

[52] Sofroniew M.V., Vinters H.V. (2010). Astrocytes: Biology and pathology. Acta Neuropathol..

[53] Gharbi T., Zhang Z., Yang G.Y. (2020). The Function of Astrocyte Mediated Extracellular Vesicles in Central Nervous System Diseases. Front. Cell Dev. Biol..

[54] Pei X., Li Y., Zhu L., Zhou Z. (2019). Astrocyte-derived exosomes suppress autophagy and ameliorate neuronal damage in experimental ischemic stroke. Exp. Cell Res..

[55] Bu X., Li D., Wang F., Sun Q., Zhang Z. (2020). Protective role of astrocyte-derived exosomal microRNA-361 in cerebral ischemic-reperfusion injury by regulating the AMPK/mTOR signaling pathway and targeting CTSB. Neuropsychiatr. Dis. Treat..

[56] Wu W., Liu J., Yang C., Xu Z., Huang J., Lin J. (2020). Astrocyte-derived exosome-transported microRNA-34c is neuroprotective against cerebral ischemia/reperfusion injury via TLR7 and the NF-kappaB/MAPK pathways. Brain Res. Bull..

[57] Xin H., Li Y., Liu Z., Wang X., Shang X., Cui Y., Zhang Z.G., Chopp M. (2013). MiR-133b promotes neural plasticity and functional recovery after treatment of stroke with multipotent mesenchymal stromal cells in rats via transfer of exosomeenriched extracellular particles. Stem Cells.

[58] Pascua-Maestro R., Gonzalez E., Lillo C., Ganfornina M.D., Falcon-Perez J.M., Sanchez D. (2018). Extracellular vesicles secreted by astroglial cells transport apolipoprotein D to neurons and mediate neuronal survival upon oxidative stress. Front. Cell Neurosci..

[59] Harris M.G., Hulseberg P., Ling C., Karman J., Clarkson B.D., Harding J.S., Zhang M., Sandor A., Christensen K., Nagy A. (2014). Immune privilege of the CNS is not the consequence of limited antigen sampling. Sci. Rep..

[60] Liu G., Li T., Yang A., Zhang X., Qi S., Feng W. (2023). Knowledge domains and emerging trends of microglia research from 2002 to 2021: A bibliometric analysis and visualization study. Front Aging Neurosci..

[61] Dong R., Huang R., Wang J., Liu H., Xu Z. (2021). Effects of Microglial Activation and Polarization on Brain Injury After Stroke. Front. Neurol..

[62] Jiang C.T., Wu W.F., Deng Y.H., Ge J.W. (2020). Modulators of Microglia Activation and Polarization in Ischemic Stroke. Mol. Med. Rep..

[63] Song Y., Li Z., He T., Qu M., Jiang L., Li W., Shi X., Pan J., Zhang L., Wang Y. (2019). M2 microglia-derived exosomes protect the mouse brain from ischemia-reperfusion injury via exosomal miR-124. Theranostics.

[64] Zhang D., Cai G., Liu K., Zhuang Z., Jia K., Pei S., Wang X., Wang H., Xu S., Cui C. (2021). Microglia exosomal miRNA-137 attenuates ischemic brain injury through targeting Notch1. Aging.

[65] Xie L., Zhao H., Wang Y., Chen Z. (2020). Exosomal shuttled miR-424-5p from ischemic preconditioned microglia mediates cerebral endothelial cell injury through negatively regulation of FGF2/STAT3 pathway. Exp. Neurol..

[66] Li F., Kang X., Xin W., Li X. (2022). The Emerging Role of Extracellular Vesicle Derived from Neurons/Neurogliocytes in Central Nervous System Diseases: Novel Insights Into Ischemic Stroke. Front. Pharmacol..

[67] Norris G.T., Smirnov I., Filiano A.J., Shadowen H.M., Cody K.R., Thompson J.A., Harris T.H., Gaultier A., Overall C.C., Kipnis J. (2018). Neuronal integrity and complement control synaptic material clearance by microglia after CNS injury. J. Exp. Med..

[68] Pluvinage J.V., Haney M.S., Smith B.A.H., Sun J., Iram T., Bonanno L., Li L., Lee D.P., Morgens D.W., Yang A.C. (2019). CD22 blockade restores homeostatic microglial phagocytosis in ageing brains. Nature.

[69] Zhao B., Fei Y., Zhu J., Yin Q., Fang W., Li Y. (2021). PAF Receptor Inhibition Attenuates Neuronal Pyroptosis in Cerebral Ischemia/Reperfusion Injury. Mol. Neurobiol..

[70] Yang M., Weng T., Zhang W., Zhang M., He X., Han C., Wang X. (2021). The Roles of Non-coding RNA in the Development and Regeneration of Hair Follicles: Current Status and Further Perspectives. Front. Cell Dev. Biol..

[71] Domingues H.S., Falcão A.M., Mendes-Pinto I., Salgado A.J., Teixeira F.G. (2020). Exosome Circuitry During (De)(Re)Myelination of the Central Nervous System. Front. Cell Dev. Biol..

[72] Frühbeis C., Kuo-Elsner W.P., Müller C., Barth K., Peris L., Tenzer S., Möbius W., Werner H.B., Nave K.A., Fröhlich D. (2020). Oligodendrocytes support axonal transport and maintenance via exosome secretion. PLoS Biol..

[73] Fröhlich D., Kuo W.P., Frühbeis C., Sun J.J., Zehendner C.M., Luhmann H.J., Pinto S., Toedling J., Trotter J., Krämer-Albers E.M. (2014). Multifaceted effects of oligodendroglial exosomes on neurons: Impact on neuronal firing rate, signal transduction and gene regulation. Philos. Trans. R. Soc. Lond. B Biol. Sci..

[74] Sun J., Yuan Q., Guo L., Xiao G., Zhang T., Liang B., Yao R., Zhu Y., Li Y., Hu L. (2022). Brain Microvascular Endothelial Cell-Derived Exosomes Protect Neurons from Ischemia–Reperfusion Injury in Mice. Pharmaceuticals.

[75] Hermann D.M., Popa-Wagner A., Kleinschnitz C., Doeppner T.R. (2019). Animal models of ischemic stroke and their impact on drug discovery. Expert. Opin. Drug Discov..

[76] Zhou J., Chen L., Chen B., Huang S., Zeng C., Wu H., Chen C., Long F. (2018). Increased serum exosomal miR-134 expression in the acute ischemic stroke patients. BMC Neurol..

[77] Chen Y., Song Y., Huang J., Qu M., Zhang Y., Geng J., Zhang Z., Liu J., Yang G.Y. (2017). Increased Circulating Exosomal miRNA-223 Is Associated with Acute Ischemic Stroke. Front. Neurol..

[78] Jiang S., Wu J., Geng Y., Zhang Y., Wang Y., Wu J., Lu C., Luo G., Zan J., Zhang Y. (2022). Identification of Differentially Expressed microRNAs Associated with Ischemic Stroke by Integrated Bioinformatics Approaches. Int. J. Genom..

[79] Ji Q., Ji Y., Peng J., Zhou X., Chen X., Zhao H., Xu T., Chen L., Xu Y. (2016). Increased Brain-Specific MiR-9 and MiR-124 in the Serum Exosomes of Acute Ischemic Stroke Patients. PLoS ONE.

[80] Qi Z., Zhao Y., Su Y., Cao B., Yang J.J., Xing Q. (2021). Serum Extracellular Vesicle-Derived miR-124-3p as a Diagnostic and Predictive Marker for Early-Stage Acute Ischemic Stroke. Front. Mol. Biosci..

[81] Kalani M.Y.S., Alsop E., Meechoovet B., Beecroft T., Agrawal K., Whitsett T.G., Huentelman M.J., Spetzler R.F., Nakaji P., Kim S. (2020). Extracellular microRNAs in blood differentiate between ischaemic and haemorrhagic stroke subtypes. J. Extracell. Vesicles.

[82] Song P., Sun H., Chen H., Wang Y., Zhang Q. (2020). Decreased Serum Exosomal miR-152-3p Contributes to the Progression of Acute Ischemic Stroke. Clin. Lab..

[83] Wang S., Jun J., Cong L., Du L., Wang C. (2021). miR-328-3p, a Predictor of Stroke, Aggravates the Cerebral Ischemia-Reperfusion Injury. Int. J. Gen. Med..

[84] He X.W., Shi Y.H., Zhao R., Liu Y.S., Li G.F., Hu Y., Chen W., Cui G.H., Su J.J., Liu J.R. (2019). Plasma Levels of miR-125b-5p and miR-206 in Acute Ischemic Stroke Patients After Recanalization Treatment: A Prospective Observational Study. J. Stroke Cerebrovasc. Dis..

[85] Allen L.M., Hasso A.N., Handwerker J., Farid H. (2012). Sequence-specific MR imaging findings that are useful in dating ischemic stroke. Radiographics.

[86] Wang W., Li D.B., Li R.Y., Zhou X., Yu D.J., Lan X.Y., Li J.P., Liu J.L. (2018). Diagnosis of Hyperacute and Acute Ischaemic Stroke: The Potential Utility of Exosomal MicroRNA-21-5p and MicroRNA-30a-5p. Cerebrovasc. Dis..

[87] Li D.B., Liu J.L., Wang W., Li R.Y., Yu D.J., Lan X.Y., Li J.P. (2017). Plasma Exosomal miR-422a and miR-125b-2-3p Serve as Biomarkers for Ischemic Stroke. Curr. Neurovasc. Res..

[88] Adams H.P., Bendixen B.H., Kappelle L.J., Biller J., Love B.B., Gordon D.L., Marsh E.E. (1993). 3rd Classification of subtype of acute ischemic stroke. Definitions for use in a multicenter clinical trial. TOAST. Trial of Org 10172 in Acute Stroke Treatment. Stroke.

[89] Van Kralingen J.C., McFall A., Ord E.N.J., Coyle T.F., Bissett M., McClure J.D., McCabe C., Macrae I.M., Dawson J., Work L.M. (2019). Altered Extracellular Vesicle MicroRNA Expression in Ischemic Stroke and Small Vessel Disease. Transl. Stroke Res..

[90] Otero-Ortega L., Alonso-López E., Pérez-Mato M., Laso-García F., Gómez-de Frutos M.C., Diekhorst L., García-Bermejo M.L., Conde-Moreno E., Fuentes B., de Leciñana M.A. (2021). Circulating Extracellular Vesicle Proteins and MicroRNA Profiles in Subcortical and Cortical-Subcortical Ischaemic Stroke. Biomedicines.

[91] Felekkis K., Pieri M., Papaneophytou C. (2021). Variability in the levels of exosomal miRNAs among human subjects could be explained by differential interactions of exosomes with the endothelium. IUBMB Life.

[92] Sullivan R., Montgomery A., Scipioni A., Jhaveri P., Schmidt A.T., Hicks S.D. (2022). Confounding Factors Impacting microRNA Expression in Human Saliva: Methodological and Biological Considerations. Genes.

[93] Guo L., Zhang Q., Ma X., Wang J., Liang T. (2017). miRNA and mRNA expression analysis reveals potential sex-biased miRNA expression. Sci. Rep..

[94] Sharma S., Eghbali M. (2014). Influence of sex differences on microRNA gene regulation in disease. Biol. Sex. Differ..

[95] Iacomino G., Siani A. (2017). Role of microRNAs in obesity and obesity-related diseases. Genes. Nutr..

[96] Mantilla-Escalante D.C., de las Hazas M.C.L., Gil-Zamorano J., del Pozo-Acebo L., Crespo M.C., Martín-Hernández R., del Saz A., Tomé-Carneiro J., Cardona F., Cornejo-Pareja I. (2019). Postprandial Circulating miRNAs in Response to a Dietary Fat Challenge. Nutrients.

[97] Telles G.D., Libardi C.A., Conceição M.S., Vechin F.C., Lixandrão M.E., De Andrade A.L.L., Guedes D.N., Ugrinowitsch C., Camera D.M. (2021). Time Course of Skeletal Muscle miRNA Expression after Resistance, High-Intensity Interval, and Concurrent Exercise. Med. Sci. Sports Exerc..

[98] Zhou Q., Shi C., Lv Y., Zhao C., Jiao Z., Wang T. (2020). Circulating microRNAs in Response to Exercise Training in Healthy Adults. Front. Genet..

